# A Socio-Ecological Examination of Weight-Related Characteristics of the Home Environment and Lifestyles of Households with Young Children

**DOI:** 10.3390/nu9060604

**Published:** 2017-06-14

**Authors:** Virginia Quick, Jennifer Martin-Biggers, Gayle Alleman Povis, Nobuko Hongu, John Worobey, Carol Byrd-Bredbenner

**Affiliations:** 1 Department of Nutritional Sciences, Rutgers University, 26 Nichol Avenue, New Brunswick, NJ 08901, USA; jmartin@aesop.rutgers.edu (J.M.-B.); worobey@rci.rutgers.edu (J.W.); bredbenner@aesop.rutgers.edu (C.B.-B.); 2 Department of Nutritional Sciences, University of Arizona, 406 Shantz Building, 1177 E. 4th Street, Tucson, AZ 85721, USA; gpovis@email.arizona.edu (G.A.P.); hongu@email.arizona.edu (N.H.)

**Keywords:** socio-ecological model, home environment, parents, child, nutrition, diet, physical activity, sleep, obesity

## Abstract

Home environment and family lifestyle practices have an influence on child obesity risk, thereby making it critical to systematically examine these factors. Thus, parents (*n* = 489) of preschool children completed a cross-sectional online survey which was the baseline data collection conducted, before randomization, in the HomeStyles program. The survey comprehensively assessed these factors using a socio-ecological approach, incorporating intrapersonal, interpersonal and environmental measures. Healthy intrapersonal dietary behaviors identified were parent and child intakes of recommended amounts of 100% juice and low intakes of sugar-sweetened beverages. Unhealthy behaviors included low milk intake and high parent fat intake. The home environment’s food supply was found to support healthy intakes of 100% juice and sugar-sweetened beverages, but provided too little milk and ample quantities of salty/fatty snacks. Physical activity levels, sedentary activity and the home’s physical activity and media environment were found to be less than ideal. Environmental supports for active play inside homes were moderate and somewhat better in the area immediately outside homes and in the neighborhood. Family interpersonal interaction measures revealed several positive behaviors, including frequent family meals. Parents had considerable self-efficacy in their ability to perform food- and physical activity-related childhood obesity protective practices. This study identified lifestyle practices and home environment characteristics that health educators could target to help parents promote optimal child development and lower their children’s risk for obesity.

## 1. Introduction

The high prevalence of obesity, especially among young children, continues to be of great public health concern given obesity’s long-term negative health effects on child growth, development and lifelong health [1,2,3]. Research suggests the pervasiveness of obesity is at least partly due to myriad socio-ecological factors that, unlike genetic factors, may be modifiable via public health interventions [4]. The socio-ecological model considers the complex interplay between intrapersonal factors (e.g., values, self-efficacy, outcome expectations), interpersonal factors (e.g., social norms, social support), and environmental factors (e.g., physical environment related to food and physical activity availability and accessibility). Understudied socioecological factors critical to childhood obesity prevention are the weight-related aspects of the home environment and family interpersonal factors and lifestyle patterns [5,6].

The socio-ecological model is a graphic depiction of the ecological theory of a specific health behavior or outcome [4,5]. It illustrates how the health and well-being of an individual is determined by multiple influences that interact at both the macro-level and micro-level environments [7]. At the macro-level, factors such as social norms, economic policies and advertising have a more indirect influence on behaviors. Micro-level factors, such as an individual’s physical and social environment (i.e., interpersonal level) and personal factors (i.e., intrapersonal level), more directly influence behaviors. In obesity research, socio-ecological theory is conceptualized as being influenced by factors across multiple levels: individual and family characteristics, and characteristics of the home, community, and region [8]. Environments that do not support healthy weight-management behaviors (e.g., access to safe parks and sidewalks for physical activity) make it difficult for individuals to engage in behaviors that prevent, limit, or reverse weight gain. To date, obesity interventions focused on prevention of weight gain in children under 5 years of age have shown limited effectiveness in reducing or limiting weight gain [9]. A systematic review of obesity prevention interventions among preschool children suggest the failure to show an intervention effect may be partly due to the lack of focus on social and environmental factors within which diet and physical activity behaviors are enacted [10].

The currently available research on the prevention and treatment of obesity among preschool-aged children and adults highlight the importance of considering the environment [11]. The micro-level of the home is the prominent shared environment of parents and their children. Parents act as ‘gate keepers’ of the home and role models for their children; they strongly influence food and physical activity behaviors and practices that may increase or decrease their child’s obesity risk [12,13,14,15,16,17,18,19,20,21]. Additionally, physical attributes of the home environment (e.g., availability of healthy foods) and parental behaviors (e.g., parent feeding practices) have been found to be associated with preschool children’s weight-related behaviors (e.g., physical activity, dietary patterns) [22]. Prior research has suggested that a number of intra- and inter-personal factors in the home environment are also associated with children’s overweight status, such as parent overweight status [23], limited daily physical activity [24], frequent family meals [25], low household availability of fruits and vegetables [26], greater daily television viewing time [25], and less parental modeling of healthy behaviors [27].

Given the home environment (intra- and inter-personal factors) may greatly influence child obesity risk, it is critical to systematically examine these factors. Few studies have comprehensively assessed the home environment and lifestyles of parents of preschool-aged children using a socio-ecological approach with reliable and validated intrapersonal, interpersonal, and environmental measures [28], which are necessary for understanding the potential influencers of obesity risk on families with young children [29]. To expand our understanding, the objective of this study was to utilize a baseline dataset collected prior to randomization to describe the socio-ecological factors related to the obesogenic home environments of parents with preschool-aged children (2 to 5 years of age) in a program called HomeStyles [30,31,32,33].

## 2. Materials and Methods 

Details of the protocol for the HomeStyles program are reported elsewhere [34] and are described in brief in this section. The Institutional Review Board at the authors’ universities approved this study (ethical approval code is #11-294Mc). All participants gave informed consent.

### 2.1. Sample & Recruitment

Parents of preschool children (ages 2 to <6 years) who resided in the catchment areas of New Jersey and Arizona in the U.S. were recruited to participate in a program that would help them build closer family bonds and raise healthier families. Recruitment notices were distributed as flyers, posters, and/or email announcements to community centers, workplaces, schools, daycare programs, doctor’s offices, and places of worship. In-person recruiting was conducted at these sites and community events. To be eligible to participate, parents had to have at least one preschool child, be able to read and write English or Spanish at about the 4th to 5th grade level, be the key decision maker with regard to family food purchases and preparation, and have consistent access to the Internet. Recruited participants began by completing a brief online eligibility screener survey. Those who were eligible were then directed to complete the online baseline survey. The online baseline survey dataset utilized in this study was collected before parents began the intervention.

### 2.2. Instruments

#### 2.2.1. Survey Development & Implementation

Development of the online survey and implementation is described in detail elsewhere [34]. In brief, the survey included an array of valid, reliable measures assessing parent and household psychographic characteristics (e.g., personal organization, family conflict) as well as parent and child weight-related behaviors (i.e., diet, physical activity, sleep) and parent weight-related cognitions (e.g., values, self-efficacy) that are described further below. Measures were selected to yield an understanding of intrapersonal and interpersonal/social behaviors and cognitions and environmental conditions in and near participants’ homes pertaining to diet and physical activity. All measures were self-report and underwent rigorous selection or development procedures to ensure they were valid and reliable, matched the goals of HomeStyles, and acceptable and accurately interpreted by the target audience [5,6,35,36]. Prior to data collection, the survey was pre-tested (*n* = 48) to identify refinements needed to improve clarity and verify accuracy of scale scoring algorithms. The survey was also pilot tested (*n* = 550) to confirm scale unidimensionality and internal consistency, and further reviewed by a panel of experts to confirm measures were of integrity and suitability to the study purpose [6]. After undergoing rigorous testing, recruitment and implementation of the survey was conducted online over a 15-month period [34]. Parents with more than one preschool child were instructed to report data for one “target” child, defined as the child born closest to a randomly selected date specified in the survey (i.e., noon on 1 June). The measures in the survey, including scale type, number of items, possible score range, and Cronbach’s alpha (as applicable), are organized by level of the socio-ecological model as presented in Table 1.

#### 2.2.2. Sociodemographic Characteristics

Sociodemographic characteristics of the sample (e.g., parent race/ethnicity, education level, age, sex) were collected. Family socio-economic status was assessed with both the 4-item Family Affluence Scale [37,38] and annual median household income based on U.S. Census Bureau zip code data. Parents rated their own and their child’s health status (poor, fair, good, very good, or excellent) using the Centers for Disease Control and Prevention’s Health-Related Quality of Life questionnaire [39,40].

#### 2.2.3. Intrapersonal Factors

Intrapersonal measures included three scales assessing the extent of parents’ personal organization [41], their need for cognition (e.g., enjoyment of thinking) [42,43], and control of stress [44]. Intrapersonal weight-related assessments included food frequency questionnaires evaluating dietary intake (e.g., fruits and vegetables, milk, sugar-sweetened beverages, fat) [45,46,47,48,49,50], physical activity level [51,52,53], screentime [54,55,56], and sleep duration [57,58] of parents and children.

#### 2.2.4. Interpersonal/Social Factors

Interpersonal/social characteristics assessed included household chaos [41,59], family conflict [60], family support for healthy eating and physical activity, frequency of family meals [61], frequency of eating family meals in various locations (e.g., in the car) [54,62,63], and frequency with which television or other media devices were used during family meals [6,22,54]. Other interpersonal/social characteristics included appraisals of the emotional environment at family mealtime [22,62,63,64], meal planning behaviors [65], self-efficacy for preparing family meals [66], and parental modeling of healthy eating behaviors and self-efficacy for childhood obesity-protective practices [6,55,67,68,69,70]. Interpersonal/social characteristics associated with physical activity included frequency of parent and child actively playing together [6], parental modeling of physical activity [22,50,51,52,71], and parental encouragement of and self-efficacy for promoting children’s physical activity [6,22,52,67,68,72,73]. The importance and value parents placed on dietary and physical activity practices and cognitions linked to obesity prevention [66,72,74] also were evaluated.

#### 2.2.5. Environmental Factors

Food-frequency questionnaires evaluated the typical availability of fruit/vegetable juice, salty/fatty snacks, sugar-sweetened beverages, and milk in the home [46,47,50,75,76]. The availability of space and supports for physical activity inside the home, immediately outside the home (yard), and neighborhood, along with perceived neighborhood safety and frequency of outdoor active play, were appraised with the HOP-Up (Home Opportunities for Physical activity check-Up) Checklist [77]. The home media environment (i.e., media devices in the home and child bedroom [22,54,55,56], amount of daily screentime children were allowed [22,54,55,56], and total time TV was on daily [78]) served as a proxy for sedentary behavior supports.

### 2.3. Data Analysis

Descriptive statistics (means, standard deviations, percentages, actual score ranges) were computed to describe the sociodemographic characteristics of study participants and intrapersonal, interpersonal/social, and environmental factors. Internal consistency for continuous scales were also measured (when applicable) using Cronbach’s alpha. SPSS software version 24.0 (IBM Corporation, Chicago, IL, USA) was used for all analyses.

## 3. Results

Of the 1221 individuals who responded to recruitment advertisements, were eligible for the study, and gave informed consent, 489 (40%) completed the baseline survey. [34]. The mean parent age was 32.34 ± 5.71 SD years and the vast majority were female (93%). More than half were white (58%), resided in New Jersey (53%), had earned a baccalaureate degree or higher (51%), and spoke English at home (87%). Slightly more than one-third of participants did not have paid employment (36%) or worked full time (38%), with the remainder working part-time.

Most households had 1 (30%) or 2 children (42%) children less than 18 years old and at least one of these children between the ages of 2 and <6 years. The target children were approximately evenly divided by sex (48% female) and had an average age of 3.85 ± 1.05 SD years. A plurality was white (49%) and most were the biological offspring of the participating parent (91%).

Most participants lived in dual parent households (82%) and had spouses/partners who had at least some post-secondary education (78%) and worked full-time (84%). A total of 17%, 58%, and 25% were low, middle, and high family affluence level [33,34], respectively. Annual median household income was based on U.S. Census Bureau zip code data for each participant’s home (mean $63,654.84 ± 24,787.07 SD).

As displayed in Table 1, intrapersonal parent measure scores indicate that participants were somewhat disorganized personally, were fairly neutral about whether they had a need for cognition, and were able to handle most stresses. On average, parents reported good to very good health status. Intake of 100% fruit/vegetable juice and milk servings were low at slightly more than half a serving daily, which was lower than the nearly three-quarters of a serving of sugar-sweetened beverages daily intake. Fat intake exceeded one-third of total daily calories. Overall, physical activity level was low while sedentary screentime was high (~6 h/day).

Child intake of fruit/vegetable juice, milk, and sugar-sweetened beverages all equaled less than one serving per day. Physical activity was moderate while sedentary screentime was high (~5 h/day). Sleep averaged 7 h nightly for parents (~62% meeting sleep recommendations of 7 or more hours per night) and total sleep duration for children (daytime naps and nighttime sleep) was nearly 11 h (62% meeting sleep recommendations (11–14 h/day for 2 year olds; 10–13 h/day for 3–5 year olds).

Psychographic household measures indicated that participants’ had somewhat chaotic households and tended to feel their families got along fairly well, disagreeing that they had family conflict. Family meals were eaten nearly twice per day and were eaten at a dining table more often than other locations. On average, TV was watched during family meals or while snacking on half the days in a week. Parents agreed that family meals had a positive emotional environment. They somewhat agreed that they planned family meals, had self-efficacy for preparing family meals, modeled healthy eating behaviors to children, and had self-efficacy for food-related childhood obesity-protective practices. With regard to physical activity, parents agreed that they encouraged children to be physically active, but actively played with children or modeled physical activity to children less than half of the days in a week. Parents strongly agreed that healthy eating and physical activity behaviors lead to positive outcomes, however the value placed on modeling healthy physical activity behaviors tended to be somewhat neutral.

The household food environment provided about 3 servings of 100% fruit/vegetable juice and 1.5 servings of sugar-sweetened beverages per household member per week. Approximately 7 servings of both milk and salty/fatty snacks were available weekly per person. Physical activity space and supports for children inside the home were moderate, with outdoor/yard and neighborhood space and supports for physical activity ratings being higher. Neighborhood safety ratings tended to be neutral, and participants reported the frequency of child active play outdoors occurred 2 to 3 times per week. Households were replete with ‘inactive’ media devices, and the time spent with these devices equaled about 8 h daily.

## 4. Discussion

Healthy intrapersonal behaviors identified in this study population include parent and child intakes of 100% fruit/vegetable juice that mirror recommendations of 4 to 6 ounces per day [80,81], with these intake levels corroborated by the household environment’s availability of 100% fruit/vegetable juice servings/household member/week. Another positive feature is the intake and household availability of sugar-sweetened beverages (e.g., soft drinks, fruit drinks) were fairly low, contributing only about 90 and 29 calories (and 18 and 6 grams of sugar) to parent and child daily intake, respectively; values which are lower than the per capita intakes found in nationally representative studies [82]. An area in great need of improvement is milk intake and availability in the household, which were far below recommendations for both parents and children [80], thereby potentially placing parents at risk of osteoporosis [83] and children at risk for decreased bone mineralization and associated sequelae [83]. These low milk intakes during childhood are especially worrisome given that milk intake tends to drop off as children, especially females, enter adolescence [82]. Also of concern are the household availability of more than 1 serving/person daily of salty/fatty snacks and the percentage of total calories contributed by fat to parents’ diets. Indeed, parents’ fat intake exceeded the upper limit of the Acceptable Macronutrient Distribution Ranges (AMDRs) [84] and was somewhat higher than the mean intake of U.S. adults [85].

Physical activity levels, time spent in sedentary activity, and the physical activity and media environment were found to be less than ideal. Much like in national reports [85], adults in this study had limited physical activity, scoring less than one-third of the maximum score possible. One-third of parents reported walking at least 10 minutes continuously and/or engaging in moderate exercise at least 5 times per week, but only 10% engaged in vigorous activity 5 or more times per week. Children had more physical activity, but achieved only about 60% of the highest score possible. Unlike parents, half of the children walked at least 10 minutes continuously, two-thirds engaged in moderate exercise, and 40% received vigorous exercise at least 5 times per week. Environmental supports for active play inside homes were moderate. That is, children had restricted space inside homes to vigorously play (e.g., the amount of active play space for half of the children was insufficient for doing more than 3 continuous somersaults or cartwheels before hitting furniture), few toys that helped them be active inside (37% had less than 5 toys supporting active play inside the home), and engaged in active play inside the home few days per week (one-third actively played indoors less than 3 times weekly). Supports and space for physical activity outdoors and in the neighborhood were higher than indoors. Almost all parents agreed or strongly agreed that the yard or area immediately outside their homes had plenty of room for kids to play games, and more than 8 out of 10 agreed or strongly agreed that there were outdoor areas like parks, pools, and playgrounds nearby where their children could play. However, the frequency of playing outdoors averaged less than 3 to 4 times weekly. (Data were collected year round from both New Jersey and Arizona, hence seasonality should not be an influence on this frequency). The relatively infrequent outdoor play may reflect the young age of the children studied and their need for adult supervision as well as the fairly neutral ratings parents gave their neighborhood for being safe from crime and biting insects and animals.

Parents and children reported 5 to 6 h of daily screentime; children exceeded the 2016 recommendations from the American Academy of Pediatrics by 5 times [86]. The home media environment was clearly conducive to sedentary behavior—children were allowed to watch television or use ‘inactive’ media devices nearly 8 h daily and television was on for 2 h, even when no one was watching. Despite recommendations to make children’s bedrooms media free [86], 56% of children had at least one media device in their bedrooms.

Adequate sleep appears protective against excess weight gain [87,88,89,90,91,92]. The nightly sleep duration recommendation for adult is 7 to 9 h per night [93]. Nearly two-thirds of parents surveyed met these recommendations, while the remainder got less than the recommendations. The mean sleep time for parents in this study, however, is higher than the 6 h and 31 min average nightly duration for U.S. adults [94]. A comparison of children’s sleep with age-specific recommendations [93] indicated that 28% got less daily sleep than recommended for their age group.

Measures of interpersonal or family social interactions indicated several positive behaviors. For example, frequent family meals eaten in a positive emotional environment without distractions, such as television and angry discussions, are associated with healthier dietary intakes [87,95,96,97,98,99,100,101]. Parents in this study reported that their families ate together almost twice daily and mealtimes were fairly calm (e.g., low stress, infrequent arguments). Most meals were eaten at a dining or kitchen table, a location associated with fewer problems with child behaviors at mealtime [102], which likely contributed to the positive emotional atmosphere reported. However, television and media devices were used fairly often while eating, and meals were eaten in front of the television more than two days per week.

Although observational learning is an important way that children learn [103,104], parents were neutral about whether they modeled healthy eating to their children. Scores on scales assessing parent modeling of healthy physical activity behaviors, sedentary activity behaviors, and active play with their children indicated that they exhibited these behaviors fewer than three days per week. Parents tended to agree that they valued modeling physical activity and not modeling sedentary behavior to children. Opportunities for parents to learn how to put these values into action are warranted.

Outcome expectations and self-efficacy are key predictors of behavior [104,105,106]. Parents were firm in their beliefs that healthy eating and physical activity improved health. Their self-efficacy scores for engaging in food-related and physical activity-related childhood obesity protective practices showed that they were confident to very confident in their ability to perform these practices. Providing opportunities for parents to increase their self-efficacy to be “very confident” could help them increase implementation of these childhood obesity protective behaviors.

Although this is one of few studies that has comprehensively assessed obesity-related factors associated with home environment and lifestyle practices among parents of preschool aged children using a socio-ecological approach, findings should be interpreted in the light of study limitations. The cross-sectional study design does not allow for inference of causality in the observed associations. Additionally, the study sample only included parents of preschool-aged children in two geographical areas of the U.S., so findings may not be generalizable to families with children of different ages or living in other areas of the country including geography (rural vs. urban). There also is a potential for self-selection bias as participants were recruited for a behavioral intervention. Lastly, all information from participants was self-reported and may be subject to both reporting error and bias. Future research should examine the relationship of socioecological factors related to the home obesogenic environment with child weight status to determine factors predictive of childhood obesity.

## 5. Conclusions

In conclusion, this study identified socioecological factors related to the obesogenic home environment of parents with preschool-aged children that could be improved to promote optimal child development while lowering the risk of childhood obesity. However, parents had a constellation of characteristics that likely would make it a challenge for them to orchestrate changes to weight-related characteristics of their home environments and lifestyles. That is, parents indicated they tended to be disorganized (e.g., late for appointments, put off chores, not dependable) and did not enjoy dealing with situations requiring a lot of thinking. Additionally, they reported high stress levels—on at least half the days in a week they felt unable to control important things in their life and felt difficulties were piling up so high they could not overcome them. They also reported households were somewhat chaotic (i.e., a real “zoo”, noisy). On a positive note, these families had low family conflict (e.g., fighting, criticizing). These findings suggest that obesity prevention interventions for parents of preschool children need to address not only obesity-protective behaviors (e.g., diet, physical activity, sleep, parent behavior modeling) and cognitions associated with behavior change (e.g., self-efficacy, values), but also should take into consideration behavioral characteristics (e.g., parent organizational skills, need for cognition, stress, household organization) that may affect their ability to realize the benefits of the intervention.

## Figures and Tables

**Table 1:** HomeStyles Study Measures and Baseline Scores (*n* = 489).

Measures	# of Items	Scale Type	Possible Score Range	Cronbach’s Alpha	Mean ± SD	Actual Score Range
**Intrapersonal Factors**						
***Parents***						
Personal Organization [41]	4	5-point agreement rating ^A^	1–5	0.64	2.60 ± 0.94	1–5
Need for Cognition [42,43]	1	5-point agreement rating ^A^	1–5	*	3.39 ± 0.97	1–5
Control of Stress [44]	2	4-point frequency rating ^B^	1–4	0.76	3.39 ± 0.76	1–4
Health Status [37,38]	1	5-point excellence rating ^C^	1–5	*	3.45 ± 0.94	1–5
100% Fruit/Vegetable Juice (servings/day) [45,47,48,49,50]	2	9-point servings drank scale ^D^	0–2.3		0.57 ± 0.57	0–2.29
Milk (servings/day) [45,47,48,49,50]	1	9-point servings drank scale ^D^	0–8	*	0.57 ± 0.45	0–1.14
Sugar-sweetened Beverages [46,50] (servings/day)	4	9-point servings eaten scale ^D^	0–4.6	*	0.71 ± 0.81	0–4.57
% Total Calories from Fat [45,47,48,49]	17	5-point servings eaten scale ^E^	0–100	*	36.66 ± 6.00	22.10–56.90
Physical Activity Level [51,52,53]	3	8-point exercise scale ^F^	0–42	*	13.8 ± 9.87	0–42
Screentime [6,56] (minutes/day)	1	minutes/day	0–1440	*	354.69 ± 279.54	0–1425
Sleep Duration (minutes/day) [57,58] ¥	1	hours/day	0–24	*	7.08 ± 1.21	4–12
***Children***						
Health Status [37,38]	1	5-point excellence rating ^C^	1–5	*	4.36 ± 0.77	2–5
Fruit/Vegetable Juice (servings/day) [45,46,47,48,49,50,76]	2	9-point servings drank scale ^D^	0–2.3	*	0.75 ± 0.56	0–2.29
Milk (servings/day) [45,46,47,48,49,50,76]	1	9-point servings drank scale ^D^	0–8	*	0.85 ± 0.35	0–1.14
Sugar-sweetened Beverage (servings/day) [45,46,47,48,49,50,76]	2	9-point servings drank scale ^D^	0–2.3	*	0.28 ± 0.42	0–2.29
Physical Activity Level [51,52,53]	3	8-point Exercise scale ^F^	0–42	*	25.83 ± 11.53	0–42
Screentime minutes/day [6,56]	1	minutes	0–1440	*	294.57 ± 261.98	
Sleep Duration (hours/day) [57,58] †	1	hours	0–24	*	10.68 ± 1.41	8–15
**Interpersonal/Social Factors**						
Household Chaos [41,57]	2	5-point agreement rating ^A^	1–5	0.86	2.51 ± 1.07	1–5
Family Conflict [60]	5	5-point agreement rating ^A^	1–5	0.86	1.99 ± 0.76	1–5
Family Support for Healthy Eating and Physical Activity [61]	4	5-point frequency rating ^G^	1–5	0.79	3.55 ± 1.35	1–5
***Food-Related***						
Family Meal frequency/week [61]	3	0–7 days for breakfast, lunch, dinner; score is sum of 3 meals	0–21	*	12.40 ± 5.02	0–21
*Family Meal Location* [54,62,63]						
In Car (days/week)	1	0–7 days	0–7	*	0.55 ± 1.4	0–7
At Fast Food Restaurant (days/week)	1	0–7 days	0–7	*	0.88 ± 1.27	0–7
At Dining Table (days/week)	1	0–7 days	0–7	*	4.64 ± 2.5	0–7
In Front of TV (days/week)	1	0–7 days	0–7	*	2.36 ± 2.52	0–7
Media Device Use at Family Meals [6,22,52] (days/week)	1	0–7 days	0–7	*	1.69 ± 2.4	0–7
TV Use at Family Meals & Snacking Occasions [6,22,52] (days/week)	1	0–7 days	0–7	*	3.50 ± 2.67	0–7
Family Mealtime Emotional Environment [22,63]	2	5-point agreement rating ^A^	1–5	0.62	4.06 ± 0.85	1–5
Family Meals are Planned [65,66]	2	5-point agreement rating ^A^	1–5	0.77	3.38 ± 1.02	1–5
Parent Family Meal Preparation Self-Efficacy [66]	2	5-point agreement rating ^A^	1–5	0.56	3.95 ± 0.9	1–5
Parent Modeling of Healthy Eating [55,69,70]	4	5-point agreement rating ^A^	1–5	0.74	3.61 ± 0.81	1–5
Parent Self-efficacy for Food-Related Childhood Obesity-Protective Practices [6,67,68]	6	5-point confidence rating ^H^	1–5	0.81	3.78 ± 0.71	1–5
***Physical Activity-Related***						
Parent: Child Co-Physical Activity (days/week) [6]	2	8-point modeling scale ^I^	0–7	0.68	3.64 ± 1.85	0–7
Parent Modeling of Physical Activity (days/week) [22,54,55,70]	2	8-point modeling scale ^I^	0–7	0.59	3.20 ± 1.31	0–6.67
Parent Modeling of Sedentary Activity (days/week) [22,54,55,70]	2	8-point modeling scale ^I^	0–7	0.72	3.51 ± 2.33	0–7
Parent Encouragement of Child Physical Activity [6,22,55,72,73]	5	5-point agreement rating ^A^	1–5	0.85	4.02 ± 0.67	1–5
***Parent Self-Efficacy for Physical-Activity Related Childhood Obesity-Protective Practices*** [6,67,68]	3	5-point confidence rating ^H^	1–5	0.84	3.49 ± 1.01	1–5
***Parent Values Related to Obesity-Protective Practices***						
Healthy Eating Outcome Expectations [66,74]	6	5-point agreement rating ^A^	1–5	0.92	4.55 ± 0.54	2–5
Physical Activity Outcome Expectations [66,74]	6	5-point agreement rating ^A^	1–5	0.94	4.44 ± 0.61	2–5
Value Placed on Modeling Physical Activity [6,22,71,72,73]	2	5-point agreement rating ^A^	1–5	0.73	3.86 ± 0.86	1–5
Valued Placed on Not Modeling Sedentary Behavior [6]	1	5-point agreement rating ^A^	1–5	*	3.81 ± 0.97	1–5
Value Placed on Physical Activity for Children [72,73]	2	5-point agreement rating ^A^	1–5	0.71	3.70 ± 0.88	1–5
**Environmental Factors**						
***Household Food Availability*** [46,47,50,75,76]						
100% Fruit/Vegetable Juice (servings/household member/week)	2	9-point servings scale ^J^	0–8	*	3.19 ± 2.06	0–8
Salty/fatty snacks (servings/household member/week)	4	9-point servings scale ^J^	0–32	*	7.9 ± 7.19	0–32
Sugar-sweetened Beverages (servings/household member/week)	4	9-point servings scale ^J^	0–8	*	1.62 ± 1.8	0–8
Milk (servings/household member/week)	1	9-point servings scale ^J^	0–8	*	6.60 ± 2.04	0–8
***Physical Activity Environment*** [77]						
Indoor Home Space & Supports For Physical Activity	6	Varies by item; 2 items are counts; 1 item is a 5-point agreement rating; ^A^ 3 items are 5-point occurrence ratings ^K^	1–5	0.74	3.31 ± 0.87	1–5
Outdoor/Yard Space & Supports For Physical Activity ^‡^	4	5-point agreement rating ^A^	1–5	0.75	4.31 ± 0.7	1–5
Neighborhood Space & Supports For Physical Activity ^§^	4	5-point agreement rating ^A^	1–5	0.88	4.01 ± 0.99	0.50–5
Neighborhood Environment Safety	2	5-point agreement rating ^A^	1–5	0.42	3.41 ± 0.87	1–5
Frequency of Active Play Outdoors	2	5-point occurrence ratings ^K^	1–5	0.54	2.55 ± 0.97	1–5
***Media Environment***						
Total Number of Inactive Media Devices (including TV) in the Home [22,54,55]	6	Total devices ^L^	0–66	*	10.51 ± 4.53	1–27
Total Number of Inactive Media Devices (including TV) in Children’s Bedrooms	7	Total # of media device types ^M^	0–7	*	1.32 ± 1.57	0–7
Time Children are Allowed to Watch TV/Movies & Use Inactive Media Devices (e.g., computers, tablets, smart phones) [6] (minutes/day)	1	minutes	0–1440	*	475.98 ± 701.11	0–1440
Total Time TV is on When No One is Watching [6,22,78] (minutes/day)	1	minutes	0–1440	*	130.18 ± 214.95	0–1410

* Not applicable. ¥ *n* = 477. † *n* = 448. ^‡^ *n* = 437. ^§^ *n* = 482. ^A^ 5-point Agreement Rating: strongly disagree, disagree, neither agree nor disagree, agree, strongly agree; scored 1 to 5 respectively with scoring reversed for negatively worded statements; scale score equals average of item scores; higher scale score indicates greater expression of the trait. ^B^ 4-point Frequency Rating: not at all, several days, more than half the days, nearly every day; scored 1 to 4; higher score indicates greater frequency. ^C^ 5-point Excellence Rating: poor, fair, good, very good, excellent; scored 1 to 5 respectively; higher score indicates better health. ^D^ 9-point Beverage Servings Rating: <1 time/week, 1 day/week, 2 days/week, 3 days/week, 4 days/week, 5 days/week, 6 days/week, 7 days/week, >1 time/day; scored 0 to 8 respectively; higher score indicates greater frequency. ^E^ 5-point Fatty Food Servings Rating: 1 time/month or less, 2 to 3 times/month, 1 to 2 times/week, 3 to 4 times/week, 5 or more times/week; scored 0 to 4 respectively; scale scoring algorithm is protected by copyright and described in detail elsewhere [79]; higher score indicates greater intake. ^F^ 8-point Exercise Days/week: 0, 1, 2, 3, 4, 5, 6, and 7; days/week weighted by exercise intensity (weights of 1, 2, 3 for walking, moderate, and vigorous activity, respectively) and summed to create scale score; higher scale score indicates greater activity level. ^G^ 5-point Frequency Rating: never, rarely, sometimes, most of the time, always; scored 1 to 5; higher score indicates greater frequency. ^H^ 5-point Confidence Rating: not at all confident, not confident, confident, quite confident, very confident; scored 1 to 5 respectively; higher scale score indicates greater confidence. ^I^ 8-point Modeling Days/week: 0 (almost never), 1, 2, 3, 4, 5, 6, and 7; days averaged to create scale score; higher score indicates more frequent modeling. ^J^ 9-point Household Servings Rating: <1 time/week, 1 day/week, 2 days/week, 3 days/week, 4 days/week, 5 days/week, 6 days/week, 7 days/week, >1 time/day; scored 0 to 8 respectively; higher score indicates greater frequency. ^K^ 5-point Occurrence Rating: almost never, 1-2 times/week; 3 to 4 times/week, 5 to 6 times/week, every day; scored 1 to 5 respectively; scale score equals average of item scores; higher scale score indicates greater occurrence of behavior. ^L^ 11-point Media Device Count: 1 = 1 to 10 = 10, 11 = more than 10; scale score equals sum of items; higher score indicates greater number of media devices. ^M^ Response for each media device in child’s bedroom was 0 = no and 1 = yes; scale score equals sum of items; higher score indicates greater number of different media device types.
